# Serum anti-lipid antibodies in patients affected by leprosy in a high-burden municipality in Brazil: a cross-sectional study

**DOI:** 10.1590/S1678-9946202567024

**Published:** 2025-04-04

**Authors:** Humberto Baptista Costa, Filipe Rocha Lima, Igor Gabriel Meneses Lima, Sávio Breno Pires Brito, Julia Bitencourt, Sérgio Arruda, Iukary Takenami

**Affiliations:** 1 Universidade Federal do Vale do São Francisco, Laboratório de Estudos Aplicados à Saúde, Paulo Afonso, Bahia, Brazil; 2 Universidade de São Paulo, Faculdade de Medicina de Ribeirão Preto, Centro de Referência Nacional em Dermatologia Sanitária e Hanseníase, Laboratório de Estudos da Pele e Modelos Alternativos, Ribeirão Preto, São Paulo, Brazil; 3 Universidade de São Paulo, Faculdade de Medicina de Ribeirão Preto, Departamento de Bioquímica e Imunologia, Ribeirão Preto, São Paulo, Brazil; 4 Serviço Nacional de Aprendizagem Industrial, Instituto de Tecnologia em Saúde, Centro Integrado de Manufatura e Tecnologia, Salvador, Bahia, Brazil; 5 Fundação Oswaldo Cruz, Instituto Gonçalo Moniz, Laboratório Avançado de Saúde Pública, Salvador, Bahia, Brazil; 6 Universidade Estadual da Bahia, Departamento de Ciências da Vida, Salvador, Bahia, Brazil

**Keywords:** Leprosy, Anti-lipid antibodies, Serological tests, Diagnosis

## Abstract

Early diagnosis plays a pivotal role in breaking the epidemiological chain of *Mycobacterium leprae* transmission. Currently, diagnosis relies on clinical, dermato-neurological features, and histological/microbiological assessments. This prospective cross-sectional study investigated whether IgA, IgM, and IgG anti-lipid antibodies can be used to improve the diagnostic performance for leprosy-affected patients in a high-burden municipality in Brazil. Serum samples from 91 volunteers, including patients with leprosy (n=62), household contacts (n=21), and endemic controls (n=8) were screened by enzyme-linked immunosorbent assays (ELISA) for IgA, IgM, and total IgG against four lipids—namely, cardiolipin (CL), phosphatidylcholine (PTC), phosphatidylethanolamine (PE), and phosphatidylinositol (PI)—and a glycosphingolipid—sulfatide (SL)—found in the bacterial cell wall. Antibodies against all lipids were detected in the sera of patients with leprosy. Significantly higher levels of IgA anti-CL, anti-PE, and anti-PTC, IgM anti-CL, and total IgG anti-PTC were observed in these patients compared to household contacts and endemic controls (p < 0.0001). ROC curve analyses demonstrated high accuracy in discriminating patients with leprosy from the contacts, with moderate to high sensitivity and specificity, even in paucibacillary patients. Despite the small study population and the absence of patients with other dermatological lesions for differential diagnosis, these findings suggest the potential of anti-lipid antibodies as biomarkers for leprosy detection. This approach offers a promising method to improve early diagnosis in high-burden areas, such as the studied municipality in Brazil.

## INTRODUCTION

Leprosy is a chronic infectious disease caused by *Mycobacterium leprae*, primarily affecting the skin and nerves, and remains a global health challenge due to difficulties in early diagnosis and disease control. Current diagnostic practices rely predominantly on clinical and histopathological assessment, which are often inconclusive due to the disease's wide spectrum of manifestations and lack of concordance among bacteriologic, histopathologic, and immunologic features^1^. This limitation is particularly evident in the identification of paucibacillary (PB) patients, in which diagnostic accuracy remains suboptimal^2-3^.

While serological tests targeting antigens such as phenolic glycolipid antigen-I (PGL-I) and leprosy IDRI diagnostic-1 (LID-1) have been developed, they primarily identify multibacillary (MB) patients and are inconsistent for PB cases^4-5^. Consequently, these methods are not recommended for routine use by World Health Organization (WHO)^6^.

Advancements in lipidomics have identified specific lipids within the *M. leprae* cell wall as promising diagnostic biomarkers^7-9^. These lipids, including cardiolipin (CL), phosphatidylcholine (PTC), phosphatidylethanolamine (PE), phosphatidylinositol (PI), and sulfatide (SL), may exhibit significant immunogenic properties and hold potential as targets for more reliable serological tests.

Previous studies have demonstrated the presence of antibodies against CL in patients with leprosy^10-12^, with evidence of their persistence throughout disease progression and even post-treatment^13^. Despite these findings, the diagnostic utility of these antibodies—particularly in differentiating PB and MB leprosy—remains insufficiently investigated. Moreover, the potential diagnostic role of antibodies against other lipids, such as PE, PI, PTC, and SL, in patients with leprosy has not been explored.

Focusing on lipids present in the *M. leprae* cell wall, this research aims to explore a potential diagnostic method that could be further refined to enhance accuracy, particularly in high-burden municipalities in Brazil.

## MATERIALS AND METHODS

### Study design and location

A prospective cross-sectional study was conducted at Couto Maia Hospital, a main referral center for leprosy treatment located in Salvador city, the capital of the Bahia State, Brazil. In 2022, the total population of Salvador city was estimated at 2.418 million inhabitants and the annual leprosy detection rate was 11.79 cases per 100,000 inhabitants, a rate considered to be of high endemicity according to national parameters^14-15^.

### Study participants

Participants were recruited via convenience sampling, including newly diagnosed leprosy patients (LP) at the hospital and healthy controls from leprosy-endemic communities. Inclusion criteria for the LP group required a confirmed diagnosis of leprosy following WHO operational classification, which considers both the bacterial index (BI) and the number of skins lesions^1^. Thus, patients with a BI of zero or fewer than five skin lesions were classified as having paucibacillary (PB) leprosy. Conversely, patients with a positive BI and/or the presence of six or more skin lesions were classified as having multibacillary (MB) leprosy^16^. The BI was assessed by skin smears collected from cutaneous lesions of the patients, following established diagnostic protocols for leprosy. These smears were reviewed at the time of diagnosis by the reference hospital's laboratory, as documented in the patients’ medical records.

Control groups included healthy household contacts (HHC) of patients with leprosy—defined as individuals who had lived in the same household as a LP for at least six months—and endemic controls (EC), healthy individuals residing in the same endemic geographical area but with no history of contact with LP or their HHC.

To mitigate potential biases introduced by non-randomized sampling, EC were selected from the same geographical area to minimize potential confounding factors related to environmental or socio-demographic characteristics. Similarly, HHC were included as they represent individuals with documented exposure to LP but did not develop the disease. Serum samples from healthy controls were collected based on outpatient care demand, ensuring the exclusion of both a leprosy diagnosis and any history of the disease. This study excluded participants with known HIV infection, those undergoing immunosuppressive therapy for autoimmune diseases, transplantation, and/or other conditions, such as cancer, to minimize confounding effects on the immune response.

All participants signed an informed consent form. The study was approved by Ethics Committee of the Gonçalo Moniz Research Center in Salvador city, Brazil (CAAE Nº 23505513.6.0000.0040).

### Samples and enzyme-linked immunosorbent assay (ELISA)

In total, five milliliters of whole blood were collected from the participants by venous puncture. Serum samples were separated from the whole blood by centrifugation. Serological analysis for IgA, IgM, and total IgG antibodies against the anti-lipid were performed as described elsewhere^17^. The lipids CL, PE, PI, and PTC were obtained from Sigma-Aldrich (Saint Louis, MI, USA), whereas glycosphingolipid SL was acquired from Avanti Polar Lipids, Inc. (Alabaster, AL, USA). All lipids were diluted to 10 µg/ml with anhydrous ethanol. Then, 50 µL of the solution was added to each well of polystyrene ELISA plates. Next, the plates were dried overnight at room temperature. The coating protocol was performed as previously described^17-20^. Then, the coated wells were washed once with 1x phosphate-buffered saline (1x PBS) at pH 7.4, blocked with 100 µL of 3% low fatty acid bovine serum albumin (3% BSA/PBS), and incubated for 1 h at room temperature. Subsequently, the plates were washed twice with 300 µL 1x PBS, and 100 µL of the serum sample diluted 1:100 in 3% BSA/PBS was added. After incubation for 1 h at room temperature, the serum samples were removed, and the plates were washed three times with 1x PBS. In sequence, 100 µL of goat-derived anti-human IgM (1:10,000), total IgA (1:10,000), and total IgG (1:50,000) labelled with horseradish peroxidase (Sigma-Aldrich) diluted in 3% BSA/PBS were added. After 1 h of incubation, a new cycle of washes was performed, and 100 µL/well of the chromogenic substrate tetramethylbenzidine was added. The reaction was stopped with 100 µL of 2N sulfuric acid. The reactions were read within 10 min at 450 nm in a spectrophotometer (Thermo Fisher Scientific, Waltham, MA, USA). Samples were not provided to two groups of three wells in each ELISA plate. Results were recorded as the average optical density (OD) of triplicate samples. If a coefficient of variance greater than 10% was observed, the samples were re-run. According to Lima *et al*.^17^, all assays included negative control samples from healthy individuals with no history of leprosy diagnosis or contact with patients, as well as positive controls from patients diagnosed with the disease. Additionally, blank wells—lacking specific antibodies but containing a peroxidase-linked secondary antibody for each tested immunoglobulin—were used to assess non-specific binding, background signal, and reagent variability. The optical density values from blank wells were subtracted from the corresponding test sample results to ensure accuracy^17^. These controls enabled the proper evaluation of the analytical system's performance. Receiver operating characteristic (ROC) curve analysis was conducted using the highest likelihood ratio (LR) to optimize sensitivity and specificity, ultimately determining the best cutoff point by comparing controls and leprosy cases.

### Statistical analysis

The primary outcome was the diagnostic utility of anti-lipid antibodies for leprosy, assessed via ROC curve analysis. Diagnostic thresholds were established based on the optimal LR. The analysis included the determination of cutoff values, area under the ROC curve (AUC), sensitivity, specificity, and their respective 95% confidence intervals (CIs), along with the LR.

Non-parametric statistical tests, including the Mann-Whitney's U test and the Kruskal-Wallis's test followed by Dunn's test, were employed to analyze differences between two and three groups, respectively, along with the Z score and effect size (r). Correlations between immunoglobulin levels and the BI or skin lesion counts were assessed using Spearman's correlation. The significance level (α) was set at 0.05, meaning that results with a p-value less than 0.05 were considered statistically significant. Statistical analysis was performed using the GraphPad Prism v.9 (GraphPad Software Inc., San Diego, CA, USA).

In accordance with Bossuyt *et al*.^21^, the Standards for Reporting of Diagnostic Accuracy Studies (STARD) guidelines were followed to enhance the completeness and transparency of the diagnostic accuracy results (Supplementary Table S1).

## RESULTS

### Detection of anti-lipid antibodies among leprosy patients and non-leprosy controls

A total of 91 participants, including 62 LP (21 PB patients; 41 MB patients) and 29 non-leprosy controls (21 HHC, 8 EC) were included in the study. Table 1 shows the participants’ basic characteristics.

**Table 1:** Study population characteristics.

Characteristic		LP (N=62)	HHC (N=21)	HC (N=8)
		n (%)		
Age, years				
	Mean ± SD	43.7 ± 16.2	40.1 ± 14.4	31.9 ± 12.6
	Median (IQR 25–75%)	42 (32.0–55.0)	37 (30.0–52.0)	28 (24.0–49.5)
Sex				
	Female	36 (58.1)	18 (85.7)	4 (50.0)
	Male	26 (41.9)	3 (14.3)	4 (50.0)
Operational classification				
	Paucibacillary	21 (33.9)	-	-
	Multibacillary	41 (66.1)	-	-
Clinical form				
	Indeterminate	6 (9.7)	-	-
	Tuberculoid	12 (19.4)	-	-
	Borderline	33 (53.2)	-	-
	Lepromatous	11 (17.7)	-	-
Number of skin lesions				
	Up to 5 skin lesions	40 (64.5)	-	-
	> 5 skin lesions	22 (35.5)	-	-
Bacilloscopy				
	Negative	46 (74.2)	-	-
	Positive	10 (16.1)	-	-
	Not performed	6 (9.7)	-	-

LP = leprosy patients; HHC = healthy household contacts; EC = endemic controls; SD = standard deviation; IQR = interquartile range.

**Figure 1:**
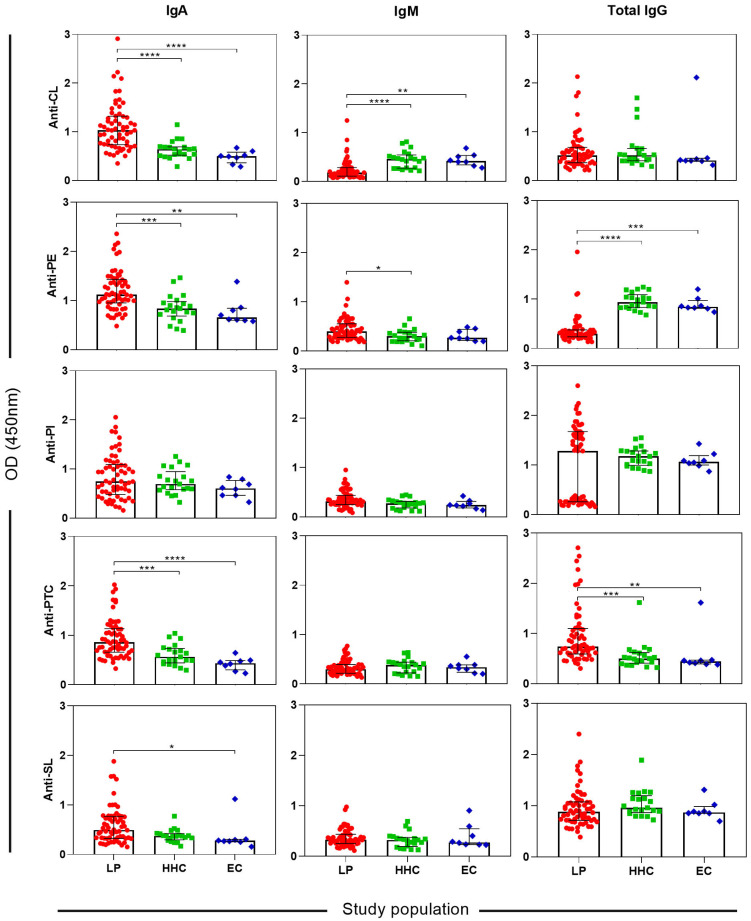
Detection of anti-CL, anti-PE, anti-PI, anti-PTC, and anti-SL IgA, IgM, and total IgG levels in LP, HHC, and EC (N=91). Multiple comparisons among groups were conducted using the Kruskal–Wallis test, followed by Dunn's post‐test for sequential pairwise comparisons. Significance levels were denoted as *, **, ***, and **** for p-values < 0.0500, < 0.0100, < 0.0010, and < 0.0001, respectively. IgA = immunoglobulin A; IgM = immunoglobulin M; IgG = immunoglobulin G; CL = cardiolipin; PE = phosphatidylethanolamine; PI = phosphatidylinositol; PTC = phosphatidylcholine; sulfatide = SL; LP = leprosy patients; HHC = healthy household contacts; EC = endemic controls.

Serum levels of IgA against CL, PE, and PTC were significantly higher in LP than in both control groups (all p < 0.0001). Furthermore, the difference in anti-SL IgA levels was also significant in LP compared to EC (p = 0.0282). IgM response to CL was higher in LP than in the non-leprosy controls (HHC, p < 0.0001; EC, p = 0.0023), whereas anti-PE was significantly higher in LP compared to HHC (p = 0.0255). Finally, IgG response to PE and PTC was significantly higher in LP than in non-leprosy controls (HHC, p < 0.0001; EC, p = 0.0003). Notably, serum levels of anti-PE IgG were significantly lower in LP compared to HHC or EC (all p < 0.0001). No difference in isotypes was observed in anti-PI (Figure 1, p > 0.0500) (Supplementary Table S2).

### Detection of anti-lipid antibodies in the clinical forms of leprosy

When we grouped the LP following WHO or Madrid classification, no difference was observed between LP in all immunoglobulin isotype and lipids (p > 0.0500) (Supplementary Tables S3 and S4). Similarly, the same occurred in LP classified by the Ridley-Jopling system. However, levels of total IgG anti-PE and anti-PI antibodies were significantly higher in patients with more advanced disease, e.g., BL and LL clinical forms (p = 0.0221, p = 0.0405, respectively) (Supplementary Table S5).

### Potential value of anti-lipid antibodies to diagnosis leprosy

**Figure 2:**
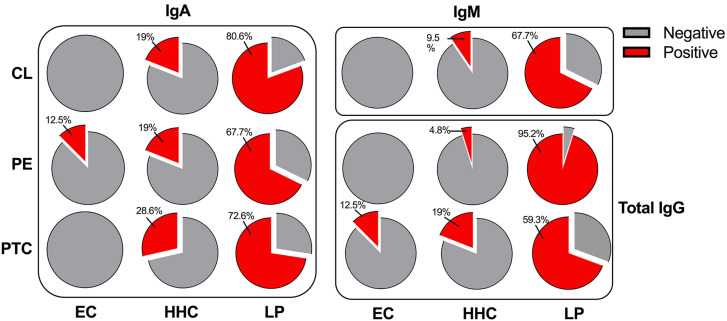
Frequency of positive results on the serological test to detect anti-CL, anti-PE, and anti-PTC IgA, IgM, and total IgG. IgA = immunoglobulin A; IgM = immunoglobulin M; IgG = immunoglobulin G; CL = cardiolipin; PE = phosphatidylethanolamine; PTC = phosphatidylcholine; SL = sulfatide; LP = leprosy patients; HHC = healthy household contacts; EC = endemic controls.

Data from LP and non-leprosy controls were used to perform the ROC curve analysis, as represented in Table 2. Based on the ROC curves, we were able to select the cutoff values for six potential markers (Figure 1). The AUC values for all six markers ranged 0.793 to 0.958. The sensitivities of the six markers for LP ranged from 67.7% to 95.2%, with specificities from 79.3% to 96.6% (Table 2). Total anti-PE IgG showed the best performance to detect LP, with a sensitivity of 95.2% and a specificity of 96.6%. Of the 62 LP, 59 (95.2%) tested positive (Figure 2). The prevalence of positive test results among PB and MB was 90.5% (19/21) and 97.6% (40/41), respectively.

**Table 2:** Comparison of receiver operating characteristic curve analysis for lipids in discriminating among patients with leprosy and non-leprosy controls.

Isotype	Lipid	AUC (95%CI)	p-value	Sensitivity % (95%CI)	Specificity % (95%CI)	LR	Cutoff
IgA	CL	0.878	<0.0001	80.6 (69.2–88.6)	86.2 (69.4–94.5)	5.85	> 0.702
	PE	0.793	<0.0001	67.7 (55.4–78.0)	82.8 (65.4–92.4)	3.93	> 1.007
	PTC	0.830	<0.0001	72.6 (60.4–82.1)	79.3 (61.6–90.2)	3.51	> 0.688
IgM	CL	0.853	<0.0001	67.7 (55.4–78.1)	93.1 (78.0–98.8)	9.82	> 0.236
IgG	PE	0.958	<0.0001	95.2 (86.7–98.7)	96.6 (82.8–99.8)	27.60	> 0.705
	PTC	0.816	<0.0001	69.4 (57.0–79.4)	82.8 (65.4–92.4)	4.02	> 0.634

AUC = area under the receiver operating characteristic curve; LR = likelihood ratio; CI = confidence interval; IgA = immunoglobulin A; IgM = immunoglobulin M; IgG = immunoglobulin G; CL = cardiolipin; PE = phosphatidylethanolamine; PTC = phosphatidylcholine.

### Correlation between anti-lipid antibodies

**Figure 3:**
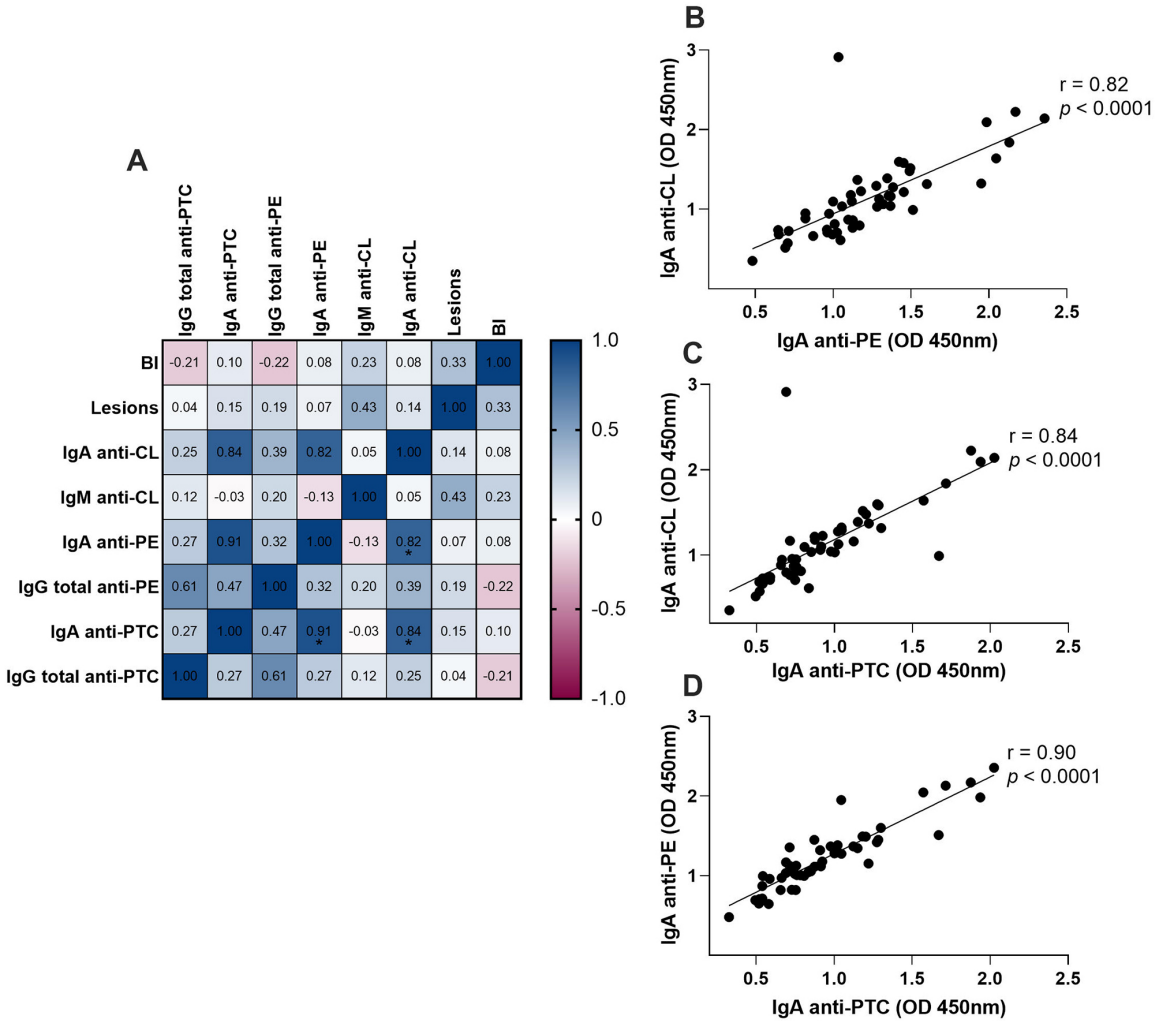
Correlation matrix (A) of the potential serological tests, bacilloscopy index and the number of lesions grouped together. Spearman's correlation coefficient is shown from −1 (pink) to 1 (blue). (B-D) Detailed of the strongest correlation observed in A. IgA = immunoglobulin A; IgM = immunoglobulin M; IgG = immunoglobulin G; CL = cardiolipin; PE = phosphatidylethanolamine; PTC = phosphatidylcholine; SL = sulfatide; BI = bacilloscopy index; LP = leprosy patients; HHC = healthy household contacts; EC = endemic controls.

We performed a matrix correlation analysis of the different variables, including anti-lipid antibodies, number of lesions, and BI, with the results being shown in color in Figure 3A. We found a significant positive correlation between the levels of anti-CL IgA and anti-PE IgA (r = 0.82, p < 0.0001), anti-PTC IgA (r = 0.84, p < 0.0001). Similarly, a strong correlation was found between serum anti-PE and anti-PTC IgA (r = 0,91, p < 0.0001). Figures 3B to 3D outline these strong correlations.

## DISCUSSION

Leprosy remains a prevalent public health challenge in Brazil, requiring the development of a straightforward and efficient diagnostic method for early detection. Early identification is crucial for disrupting the transmission chain and mitigating the physical, emotional, and social sequelae associated with the disease's progression^22^. However, despite a reduction in the annual leprosy detection rate in Brazil from 13.70 cases per 100,000 inhabitants in 2018 to 8.59 cases in 2021, contextual interpretation is essential^14^. Operational challenges such as the impact of the coronavirus disease 2019 (COVID-19) pandemic and underreporting must be considered. These factors underscore the persistent transmission of leprosy, emphasizing the need for heightened awareness and concerted efforts by government authorities.

In 2021, WHO introduced a comprehensive strategy titled "Towards zero leprosy: Global Leprosy (Hansen's Disease) Strategy 2021-2030," aiming at eliminating leprosy by achieving zero new cases, zero infection and disease, zero disability, and eradicating stigma and discrimination by 2030^1^. However, achieving these objectives hinges on advancing current diagnostic tests. Our results offer a promising alternative, serving as a straightforward, cost-effective, and highly accurate option for diagnosing leprosy. The decision to employ an indirect ELISA was influenced by the widespread availability and simplicity of the ELISA reader, reinforcing the practicality of our approach.

The anti-lipid antibodies proved incapable of distinguishing between the clinical forms of leprosy based on the Madrid and/or Ridley–Jopling classifications. MB patients with a high bacillary load, according to the operational classification, exhibit elevated IgM responses to PGL-I and both IgG responses to LID-1, as well as IgM response to the disaccharide component of NDO-LID. Conversely, PB patients display lower levels of antibody response associated with strong Th1 immune polarization and a low bacillary load^23-24^. Consequently, serological tests are anticipated to yield a more robust antibody response in MB patients. Nevertheless, our findings indicate the presence of these antibodies in all patients with leprosy, suggesting their potential utility as biomarkers for diagnostic screening to identify previously undetected cases of the disease.

The origin of anti-lipid antibodies in LP remains unclear. We propose that these antibodies may arise from activation of polyclonal B cells triggered by lipid components of *M. leprae*. Alternatively, anti-lipid antibodies could be a result of cross-reaction between mycobacterial antigens and autoantigens. This is conceivable since chronic tissue injury can expose self-epitopes within host cells, resembling epitopes encoded by mycobacteria. From that perspective, previous studies have shown that CL, PE, and phosphatidylinositol PI are often present in biological membranes. In the presence of an infectious agent, the inflammatory process induced by cell damage may expose the components, leading to the production of self-directed antibodies^25-27^.

This phenomenon is evident in antiphospholipid syndrome, in which autoantibodies consistently test positive. However, none of the LP in our study experienced thrombotic events or cardiovascular involvement, even those with elevated levels of anti-CL antibodies. This suggests that the antibody levels observed may not be sufficient to attribute them to an autoimmune process. We highlight that inhibition tests and assessments of cofactor dependence were not conducted, which could have further clarified the nature of the antibodies. Notably, the association of lipid antibodies with infectious diseases was initially identified in syphilis, but anti-CL antibodies have also been detected in tuberculosis, HIV/AIDS, hepatitis C, and, more recently, in COVID-19^17,28,29^. Collectively, these findings suggest that antiphospholipid antibodies may be relevant to the pathogenesis of leprosy, although their high prevalence remains unexplained.

Of the 15 markers assessed, six showed promise as potential serum biomarkers capable of distinguishing LP from HHC and EC. Notably, the ROC curve analyses revealed that total IgG ELISA test against PE exhibited remarkable accuracy in discriminating LP from HHC and EC, showing the highest sensitivity and specificity. Interestingly, anti-PE antibody production showed a selective reduction in LP compared to non-leprosy controls. Marques *et al*.^30^ highlighted that PE is a key lipid present in all cell wall fractions and cell membranes of *M. leprae*. Consequently, we hypothesized that the variations in antibody production could result from exposure to *M. leprae* cell wall lipids, suppressing (anti-PE antibodies of a particular class) or enhancing (anti-CL and anti-PTC) the host immune response. Considering these observations, Murray *et al*.^31^ demonstrated that the conversion of lipomannan to Man-LAM, along with other lipids, may influence the nature of host immunity in response to bacillus exposure. This supports the idea that mycobacterial cell walls could impact antibody production, as indicated by our results.

The sensitivity and specificity of our test performed satisfactorily compared to other tests based on the detection of PGL-I, NDO-LID, LID-1, and NDO-BSA. Notably, the percentage of seropositivity detected using total anti-PE IgG in LP was 95.2%, exceeding the proportions previously reported using PGL-I (64%), LID-1 (71.7%), NDO-LID (78%), and NDO-BSA (78.7%)^23^. These findings are particularly encouraging when contrasted with the diagnostic sensitivity of anti-PGL-I, which, in certain evaluations, exhibited only 30% sensitivity^32^. Moreover, our test revealed a positivity rate of only 3.4% in controls.

We highlight that the sensitivity and specificity percentages reported for the other antigens mentioned above are solely for detecting LP in the MB form and are less effective for identifying PB patients. In contrast, our tested biomarkers were capable of detecting both MB and PB patients. Additionally, the IgA response is particularly noteworthy, as this immunoglobulin also demonstrated good performance. Notably, LP showed higher levels of IgA than the controls, and the production of certain IgA antibodies appears to be positively correlated with CL, PE, and PTC, suggesting a significant activation of B lymphocytes associated with a diverse array of IgA (polyclonal IgA). Consistent with our findings, an elevated IgA response to NDO-HSA (a conjugate formed by natural octyl disaccharide bound to human serum albumin) has been previously observed across the spectrum of leprosy disease and has proven to be a valuable complementary marker for MB leprosy^33^.

This study holds limitations, particularly concerning the sample size and the uneven distribution among participant groups. Although the sample was carefully selected to represent the local context of leprosy endemicity, the limited number of endemic controls reduces statistical power and the ability to detect smaller effect sizes. Additionally, the small sample size increases the risk of Type I and Type II errors, which limits the generalizability of the findings to populations or settings outside Salvador city, Bahia State. The use of a convenience sample for non-leprosy controls also introduces the potential for selection bias, affecting the comparability of groups. Given that the study aimed to explore diagnostic markers in a high-endemicity context, the conclusions should be interpreted with caution, and future studies using randomized designs are recommended to validate and expand upon the findings.

The study did not systematically assess comorbidities or factors such as tuberculosis, syphilis, autoimmune diseases, or the use of medications unrelated to immunosuppression, all of which could potentially influence antibody levels and diagnostic performance. To enhance the robustness of cut-off determination and diagnostic metrics, future studies should include serum-based negative controls from confirmed non-leprosy individuals, as well as patients with other conditions that may affect test results. Additionally, it is crucial to investigate the role of these antibodies in the immunopathogenesis of leprosy, particularly across the various stages of the disease, such as infection, subclinical, preclinical, and reactional states, as well as their response to treatment. This study serves as an initial pilot to explore better biomarkers, laying the groundwork for future research involving diverse populations with varying endemic profiles and clinical conditions.

## CONCLUSION

Investigations into anti-lipid antibodies in leprosy patients (LP) are relatively scarce. This study is the first to explore antibodies to phosphatidylethanolamine (PE) and phosphatidylcholine (PTC) in LP. Here, we delineate the profile of anti-lipid antibodies in LP, healthy household contacts (HHC), and endemic controls (EC) as a means to assess the production of these antibodies in a highly endemic area. Using an in-house ELISA, we confirmed elevated levels of antibodies, notably anti-CL, anti-PE, and anti-PTC IgA, anti-CL IgM, and total anti-PTC IgG in the sera of LP. These findings provide evidence for the potential utility of these antibodies in aiding clinical diagnosis and serological screening of contacts.
