# Supplementary material for: Serum anti-lipid antibodies in patients affected by leprosy in a high-burden municipality in Brazil: a cross-sectional study
Source: Rev Inst Med Trop Sao Paulo. 2025 Apr 4;67:e24. doi: 10.1590/S1678-9946202567024 (PMC11984353; doi:10.1590/S1678-9946202567024)
Supplement: Supplementary file 1 [file 1678-9946-rimtsp-67-S1678-9946202567024-supp.pdf]

REVISTA DO INSTITUTO DE MEDICINA TROPICAL DE SÃO PAULO

JOURNAL OF THE SÃO PAULO INSTITUTE OF TROPICAL MEDICINE

# SUPPLEMENTARY MATERIAL

https://doi.org/10.48331/scielodata.GIP3HW

## Serum anti-lipid antibodies in patients affected by leprosy in a high-burden municipality in Brazil: a cross-sectional study

**Humberto Baptista Costa**[1], **Filipe Rocha Lima**[2,3], **Igor Gabriel Meneses Lima**[1], **Sávio Breno Pires Brito**[1], **Julia Bitencourt**[4], **Sérgio Arruda**[5,6], **Iukary Takenami**[1]

[1]Universidade Federal do Vale do São Francisco, Laboratório de Estudos Aplicados à Saúde, Paulo Afonso, Bahia, Brazil

[2]Universidade de São Paulo, Faculdade de Medicina de Ribeirão Preto, Centro de Referência Nacional em Dermatologia Sanitária e Hanseníase, Laboratório de Estudos da Pele e Modelos Alternativos, Ribeirão Preto, São Paulo, Brazil

[3]Universidade de São Paulo, Faculdade de Medicina de Ribeirão Preto, Departamento de Bioquímica e Imunologia, Ribeirão Preto, São Paulo, Brazil

[4]Serviço Nacional de Aprendizagem Industrial, Instituto de Tecnologia em Saúde, Centro Integrado de Manufatura e Tecnologia, Salvador, Bahia, Brazil

[5]Fundação Oswaldo Cruz, Instituto Gonçalo Moniz, Laboratório Avançado de Saúde Pública, Salvador, Bahia, Brazil

[6]Universidade Estadual da Bahia, Departamento de Ciências da Vida, Salvador, Bahia, Brazil

**Correspondence to:** Iukary Takenami
Universidade Federal do Vale do São Francisco, Laboratório de Estudos Aplicados à Saúde, Av. da Amizade, 1900, CEP 48607-235, Paulo Afonso, BA, Brazil
Tel: +55 71 996232211

**E-mail:** iukary.takenami@univasf.edu.br

**Received:** 18 December 2024

**Accepted:** 17 February 2025

**Supplementary Table S1** - STARD checklist.

| Section & Topic | No | Item | Reported on page # |
|---|---|---|---|
| **TITLE OR ABSTRACT** | | | |
| | 1 | Identification as a study of diagnostic accuracy using at least one measure of accuracy (such as sensitivity, specificity, predictive values, or AUC) | 1# |
| **ABSTRACT** | | | |
| | 2 | Structured summary of study design, methods, results, and conclusions (for specific guidance, see STARD for Abstracts) | 1# |
| **INTRODUCTION** | | | |
| | 3 | Scientific and clinical background, including the intended use and clinical role of the index test | 1 and 2# |
| | 4 | Study objectives and hypotheses | 2# |
| **METHODS** | | | |
| *Study design* | 5 | Whether data collection was planned before the index test and reference standard were performed (prospective study) or after (retrospective study) | 2# |
| *Participants* | 6 | Eligibility criteria | 2# |
| | 7 | On what basis potentially eligible participants were identified (such as symptoms, results from previous tests, inclusion in registry) | 2# |
| | 8 | Where and when potentially eligible participants were identified (setting, location and dates) | 2# |
| | 9 | Whether participants formed a consecutive, random or convenience series | 2# |
| *Test methods* | 10a | Index test, in sufficient detail to allow replication | 2 and 3# |
| | 10b | Reference standard, in sufficient detail to allow replication | 2 and 3# |
| | 11 | Rationale for choosing the reference standard (if alternatives exist) | 2 and 3# |
| | 12a | Definition of and rationale for test positivity cut-offs or result categories of the index test, distinguishing pre-specified from exploratory | 2 and 3# |
| | 12b | Definition of and rationale for test positivity cut-offs or result categories of the reference standard, distinguishing pre-specified from exploratory | Table 2 and 6# |
| | 13a | Whether clinical information and reference standard results were available to the performers/readers of the index test | 2 and 3# |
| | 13b | Whether clinical information and index test results were available to the assessors of the reference standard | 2 and 3# |



This is an open-access article distributed under the terms of the Creative Commons Attribution License.

**Supplementary Table S1** - STARD checklist. (cont.)

| Section & Topic | No | Item | Reported on page # |
|---|---|---|---|
| *Analysis* | 14 | Methods for estimating or comparing measures of diagnostic accuracy | 3# |
| | 15 | How indeterminate index test or reference standard results were handled | Not applicable |
| | 16 | How missing data on the index test and reference standard were handled | Not applicable |
| | 17 | Any analyses of variability in diagnostic accuracy, distinguishing pre-specified from exploratory | 3# |
| | 18 | Intended sample size and how it was determined | 2# |
| | | | |
| **RESULTS** | | | |
| *Participants* | 19 | Flow of participants, using a diagram | Not applicable |
| | 20 | Baseline demographic and clinical characteristics of participants | Table 1 and 4# |
| | 21a | Distribution of severity of disease in those with the target condition | Not applicable |
| | 21b | Distribution of alternative diagnoses in those without the target condition | Not applicable |
| | 22 | Time interval and any clinical interventions between index test and reference standard | 2 and 3# |
| *Test results* | 23 | Cross tabulation of the index test results (or their distribution) by the results of the reference standard | 7# |
| | 24 | Estimates of diagnostic accuracy and their precision (such as 95% confidence intervals) | Table 2 and 6# |
| | 25 | Any adverse events from performing the index test or the reference standard | Not applicable |
| **DISCUSSION** | | | |
| | 26 | Study limitations, including sources of potential bias, statistical uncertainty, and generalisability | 8# |
| | 27 | Implications for practice, including the intended use and clinical role of the index test | 8# |
| **OTHER INFORMATION** | | | |
| | 28 | Registration number and name of registry | Not applicable |
| | 29 | Where the full study protocol can be accessed | Not applicable |
| | 30 | Sources of funding and other support; role of funders | 8 and 9# |

**Supplementary Table S2** - Values of lipid antibodies in patients affected by leprosy, household contacts and endemic controls.

| Lipids | EC | HHC | LP | P-value* | LP vs. EC | | | LP vs. HHC | | |
|---|---|---|---|---|---|---|---|---|---|---|
| | median (IQR 25–75%). pg/mL | | | | P-value** | Z score | Effect size (r)*** | P-value** | Z score | Effect size (r)*** |
| **IgA** | | | | | | | | | | |
| CL | 0.4980 (0.3641–0.5804) | 0.6412 (0.5203–0.6909) | 1.0340 (0.7354–1.3180) | <0.0001 | <0.0001 | 4.519 | 0.66 | <0.0001 | 4.565 | 0.50 |
| PE | 0.6587 (0.5993–0.8419) | 0.8361 (0.6828–0.9790) | 1.1220 (0.9513–1.4290) | <0.0001 | 0.0008 | 3.313 | 0.39 | 0.0008 | 3.645 | 0.40 |
| PI | 0.6025 (0.4635–0.7655) | 0.6948 (0.5783–0.9457) | 0.7453 (0.4777–1.0910) | 0.3568 | >0.9999 | 1.430 | 0.17 | >0.9999 | 0.368 | 0.04 |
| PTC | 0.4368 (0.3006–0.4893) | 0.5644 (0.4420–0.7333) | 0.8589 (0.6553–1.1330) | <0.0001 | 0.0006 | 4.378 | 0.52 | 0.0006 | 3.733 | 0.41 |
| SL | 0.2756 (0.2543–0.3030) | 0.3710 (0.2936–0.4212) | 0.4941 (0.3249–0.7682) | 0.0081 | 0.1027 | 2.597 | 0.31 | 0.1027 | 2.117 | 0.23 |
| **IgM** | | | | | | | | | | |
| CL | 0.4195 (0.3407–0.5326) | 0.4581 (0.2736–0.5430) | 0.1840 (0.1078–0.2873) | <0.0001 | <0.0001 | 3.363 | 0.40 | <0.0001 | 4.749 | 0.52 |
| PE | 0.2651 (0.2144–0.4409) | 0.3024 (0.2077–0.3809) | 0.4009 (0.2760–0.5540) | 0.0147 | 0.0255 | 1.663 | 0.20 | 0.0255 | 2.631 | 0.29 |
| PI | 0.2376 (0.1804–0.3129) | 0.2730 (0.1812–0.3174) | 0.3113 (0.2485–0.4365) | 0.0251 | 0.0825 | 1.936 | 0.23 | 0.0825 | 2.204 | 0.24 |
| PTC | 0.3391 (0.2375–0.3863) | 0.3857 (0.2245–0.4416) | 0.2992 (0.2159–0.3949) | 0.5064 | 0.7683 | 0.455 | 0.05 | 0.7683 | 1.136 | 0.12 |
| SL | 0.2771 (0.2317–0.5454) | 0.3231 (0.1933–0.3743) | 0.3276 (0.2542–0.4371) | 0.6960 | >0.9999 | 0.213 | 0.02 | >0.9999 | 0.848 | 0.09 |
| **IgG** | | | | | | | | | | |
| CL | 0.4164 (0.4039–0.4578) | 0.5217 (0.4081–0.6600) | 0.5177 (0.3688–0.6843) | 0.5975 | >0.9999 | 0.976 | 0.11 | >0.9999 | 0.107 | 0.01 |
| PE | 0.8432 (0.8126–0.9724) | 0.9377 (0.8304–1.0920) | 0.2935 (0.2364–0.3770) | <0.0001 | <0.0001 | 3.884 | 0.46 | <0.0001 | 6.434 | 0.70 |
| PI | 1.0660 (0.9986–1.1880) | 1.1730 (0.9931–1.2820) | 1.2830 (0.2566–1.674) | 0.9593 | >0.9999 | 0.190 | 0.02 | >0.9999 | 0.181 | 0.02 |
| PTC | 0.4428 (0.3958–0.4715) | 0.5042 (0.4114–0.6216) | 0.7409 (0.5949–1.102) | <0.0001 | 0.0001 | 3.331 | 0.39 | 0.0001 | 4.075 | 0.44 |
| SL | 0.8684 (0.8518–0.9845) | 0.9585 (0.8629–1.1930) | 0.8816 (0.7124–1.074) | 0.1649 | 0.1763 | 0.141 | 0.01 | 0.1763 | 1.890 | 0.20 |

IgA = immunoglobulin A; IgM = immunoglobulin M; IgG = immunoglobulin G; CL = cardiolipin;PE = phosphatidylethanolamine; PI = phosphatidylinositol; PTC = phosphatidylcholine; SL = sulfatide; LP = leprosy patients; HHC = household contacts; EC = endemic controls; IQR = interquartile range; *Kruskal-Wallis test; **Kruskal-Wallis test with Dunn's multiple comparison; ***Effect size: small if r=0.10, medium if r=0.30, and large if r=0.50.

**Supplementary Table S3** - Values of lipid antibodies in patients affected by leprosy stratified by WHO classification.

| Lipids | PB | MB | P-value* | Z score | Effect size (r)** |
|---|---|---|---|---|---|
| | median (IQR 25–75%). pg/mL | | | | |
| **IgA** | | | | | |
| CL | 0.9445 (0.7081–1.1980) | 1.0940 (0.7780–1.453) | 0.2190 | 0.641 | 0.08 |
| PE | 1.0470 (0.9675–1.2790) | 1.1780 (0.8559–1.520) | 0.2304 | 0.475 | 0.06 |
| PI | 0.7439 (0.4043–1.0740) | 0.7796 (0.5375–1.104) | 0.4970 | 0.609 | 0.07 |
| PTC | 0.7281 (0.6564–0.8917) | 0.9235 (0.6223–1.274) | 0.0626 | 1.138 | 0.14 |
| SL | 0.4441 (0.3182–0.6774) | 0.5698 (0.3371–0.7820) | 0.1154 | 1.508 | 0.19 |
| **IgM** | | | | | |
| CL | 0.1710 (0.0986–0.2470) | 0.1980 (0.1120–0.3722) | 0.2134 | 1.004 | 0.12 |
| PE | 0.3793 (0.2349–0.5224) | 0.4041 (0.2853–0.5841) | 0.3832 | 0.807 | 0.10 |
| PI | 0.3442 (0.2091–0.5281) | 0.3055 (0.2532–0.3920) | 0.7593 | 0.087 | 0.01 |
| PTC | 0.3056 (0.2022–0.4123) | 0.2928 (0.2217–0.3944) | 0.8944 | 0.022 | 0.003 |
| SL | 0.2960 (0.2210–0.4036) | 0.3334 (0.2595–0.4881) | 0.2720 | 3.528 | 0.44 |
| **IgG** | | | | | |
| CL | 0.5137 (0.3517–0.6952) | 0.5217 (0.3910–0.6889) | 0.7318 | 0.116 | 0.01 |
| PE | 0.2563 (0.2082–0.3388) | 0.3041 (0.2427–0.3905) | 0.0983 | 0.862 | 0.10 |
| PI | 0.3209 (0.2261–1.6240) | 1.2930 (0.2760–1.6740) | 0.2134 | 1.185 | 0.15 |
| PTC | 0.7014 (0.5215–1.0700) | 0.7464 (0.6182–1.1200) | 0.5952 | 0.347 | 0.04 |
| SL | 0.7817 (0.5810–1.0560) | 0.9042 (0.7346–1.1330) | 0.1053 | 0.932 | 0.11 |

IgA = immunoglobulin A; IgM = immunoglobulin M; IgG = immunoglobulin G; CL = cardiolipin; PE = phosphatidylethanolamine; PI = phosphatidylinositol; PTC = phosphatidylcholine; SL = sulfatide; PB = paucibacillary; MB = multibacillary; IQR = interquartile range; *Kruskal-Wallis test; **Effect size: small if r=0.10, medium if r=0.30, and large if r=0.50.

**Supplementary Table S4A** - Values of lipid antibodies in patients affected by leprosy stratified by Ridley-Jopling classification.

| Lipids | TT | BT | BB | BL | LL | P-value* |
|---|---|---|---|---|---|---|
| | median (IQR 25–75%). pg/mL | | | | | |
| **IgA** | | | | | | |
| CL | 0.8795 (0.7119–1.274) | 0.7089 (0.6629–0.7431) | 0.7824 (0.5219–1.118) | 1.0950 (0.8678–1.3700) | 1.5170 (0.8826–1.831) | 0.0842 |
| PE | 1.0310 (0.9821–1.336) | 0.9601 (0.8719–0.9618) | 1.0380 (0.6603–1.476) | 1.1550 (0.9993–1.4520) | 1.4220 (0.8210–1.985) | 0.3599 |
| PI | 0.8121 (0.3560–1.114) | 0.3690 (0.3309–0.4011) | 0.5365 (0.2852–0.6844) | 0.9392 (0.5655–1.1290) | 1.0780 (0.6790–1.433) | 0.0272 |
| PTC | 0.7151 (0.6532–0.8452) | 0.5861 (0.5393–0.5876) | 0.8215 (0.4672–1.5330) | 0.9775 (0.7563–1.2210) | 1.1830 (0.6570–1.711) | 0.0707 |
| SL | 0.4461 (0.3233–0.7101) | 0.2479 (0.2476–0.3359) | 0.2999 (0.1982–0.5184) | 0.6478 (0.3603–0.8282) | 0.7792 (0.4888–1.003) | 0.0142 |
| **IgM** | | | | | | |
| CL | 0.1665 (0.09748–0.2758) | 0.1390 (0.08540–0.2650) | 0.1555 (0.1203–0.3415) | 0.1980 (0.1270–0.3620) | 0.2880 (0.1090–0.6190) | 0.4495 |
| PE | 0.4218 (0.2800–0.5240) | 0.2643 (0.2288–0.4041) | 0.3010 (0.2550–0.3444) | 0.4105 (0.2884–0.6057) | 0.5125 (0.2960–0.7590) | 0.0935 |
| PI | 0.3032 (0.2379–0.4297) | 0.3841 (0.1930–0.4293) | 0.2613 (0.1525–0.3658) | 0.3099 (0.2783–0.3937) | 0.3678 (0.2611–0.5858) | 0.5145 |
| PTC | 0.2990 (0.2131–0.4643) | 0.2129 (0.1927–0.4082) | 0.3221 (0.2793–0.3399) | 0.2883 (0.2166–0.4013) | 0.3463 (0.2544–0.6178) | 0.4972 |
| SL | 0.3136 (0.2447–0.4086) | 0.2597 (0.2069–0.3229) | 0.286 (0.2204–0.3261) | 0.3334 (0.2696–0.4264) | 0.4987 (0.2592–0.6077) | 0.1194 |
| **IgG** | | | | | | |
| CL | 0.5445 (0.3502–0.7141) | 0.5430 (0.2547–0.8357) | 0.4192 (0.2609–0.8530) | 0.5027 (0.3560–0.6020) | 0.7033 (0.4670–0.9120) | 0.2799 |
| PE | 0.2706 (0.2223–0.3394) | 0.2199 (0.1739–0.2363) | 0.2557 (0.2224–0.2886) | 0.3130 (0.2511–0.3871) | 0.3938 (0.3526–0.5790) | 0.0066 |
| PI | 0.8023 (0.2437–1.738) | 0.2053 (0.1900–0.2639) | 0.2464 (0.2267–0.3289) | 1.5510 (0.3092–1.6740) | 1.6050 (1.282–2.1270) | 0.0076 |
| PTC | 0.8061 (0.5980–1.735) | 2.0530 (0.6189–2.447) | 0.7170 (0.5366–0.7731) | 0.7603 (0.5794–1.1090) | 0.9557 (0.6934–0.3290) | 0.4478 |
| SL | 0.9270 (0.6937–1.061) | 0.7162 (0.5459–0.7621) | 0.7283 (0.6480–0.8686) | 0.9393 (0.7344–1.1480) | 1.1170 (0.9042–1.625) | 0.0192 |

IgA = immunoglobulin A; IgM = immunoglobulin M; IgG = immunoglobulin G; CL = cardiolipin; PE = phosphatidylethanolamine; PI = phosphatidylinositol; PTC = phosphatidylcholine; SL = sulfatide; TT = tuberculoid leprosy; BT = borderline tuberculoid; BB = borderline borderline; BL = borderline lepromatous; LL = lepromatous leprosy; IQR = interquartile range; *Kruskal-Wallis test.

**Supplementary Table S4B** - Statistical metrics for lipid antibodies in patients affected by leprosy stratified by Ridley-Jopling classification.

| Lipids | TT *vs.* BT | | | TT *vs.* BB | | | TT *vs.* BL | | | TT *vs.* LL | | | BT *vs.* BB | | |
|---|---|---|---|---|---|---|---|---|---|---|---|---|---|---|---|
| | P-value* | Z score | Effect size (r)** | P-value* | Z score | Effect size (r)** | P-value* | Z score | Effect size (r)** | P-value* | Z score | Effect size (r)** | P-value* | Z score | Effect size (r)** |
| **IgA** | | | | | | | | | | | | | | | |
| CL | >0.9999 | 1.246 | 0.32 | >0.9999 | 0.878 | 0.02 | >0.9999 | 0.862 | 0.14 | >0.9999 | 1.609 | 0.33 | >0.9999 | 0.388 | 0.14 |
| PE | >0.9999 | 0.961 | 0.24 | >0.9999 | 0.149 | 0.03 | >0.9999 | 1.072 | 0.18 | >0.9999 | 1.122 | 0.23 | >0.9999 | 0.699 | 0.26 |
| PI | >0.9999 | 1.513 | 0.39 | >0.9999 | 1.094 | 0.27 | >0.9999 | 1.089 | 0.18 | 0.9401 | 1.675 | 0.34 | >0.9999 | 0.452 | 0.17 |
| PTC | >0.9999 | 0.927 | 0.23 | >0.9999 | 0.336 | 0.08 | 0.7115 | 1.805 | 0.30 | 0.4053 | 2.048 | 0.42 | >0.9999 | 1.039 | 0.39 |
| SL | >0.9999 | 1.195 | 0.30 | >0.9999 | 0.915 | 0.22 | >0.9999 | 1.438 | 0.24 | 0.2789 | 2.199 | 0.45 | >0.9999 | 0.317 | 0.12 |
| **IgM** | | | | | | | | | | | | | | | |
| CL | >0.9999 | 0.342 | 0.08 | >0.9999 | 0.280 | 0.07 | >0.9999 | 0.843 | 0.14 | 0.9401 | 1.675 | 0.34 | >0.9999 | 0.501 | 0.18 |
| PE | >0.9999 | 1.187 | 0.30 | >0.9999 | 1.411 | 0.35 | >0.9999 | 0.433 | 0.07 | >0.9999 | 1.309 | 0.27 | >0.9999 | 0.063 | 0.02 |
| PI | >0.9999 | 0.238 | 0.06 | >0.9999 | 0.901 | 0.22 | >0.9999 | 0.267 | 0.04 | >0.9999 | 1.153 | 0.24 | >0.9999 | 0.883 | 0.33 |
| PTC | >0.9999 | 0.752 | 0.19 | >0.9999 | 0.233 | 0.05 | >0.9999 | 0.196 | 0.03 | >0.9999 | 1.173 | 0.24 | >0.9999 | 0.812 | 0.30 |
| SL | >0.9999 | 0.886 | 0.22 | >0.9999 | 0.705 | 0.17 | >0.9999 | 0.558 | 0.09 | 0.6435 | 1.850 | 0.38 | >0.9999 | 0.215 | 0.08 |
| **IgG** | | | | | | | | | | | | | | | |
| CL | >0.9999 | 0.033 | 0.01 | >0.9999 | 0.579 | 0.14 | >0.9999 | 0.492 | 0.08 | >0.9999 | 1.434 | 0.29 | 0.3475 | 0.409 | 0.15 |
| PE | >0.9999 | 1.413 | 0.36 | >0.9999 | 0.588 | 0.14 | >0.9999 | 1.247 | 0.21 | 0.0956 | 2.591 | 0.54 | >0.9999 | 0.748 | 0.28 |
| PI | >0.9999 | 1.530 | 0.39 | >0.9999 | 1.234 | 0.30 | >0.9999 | 1.093 | 0.18 | 0.3410 | 2.119 | 0.44 | >0.9999 | 0.360 | 0.13 |
| PTC | >0.9999 | 1.070 | 0.27 | >0.9999 | 0.953 | 0.23 | >0.9999 | 0.332 | 0.05 | >0.9999 | 0.620 | 0.12 | >0.9999 | 1.625 | 0.61 |
| SL | >0.9999 | 1.371 | 0.35 | >0.9999 | 1.065 | 0.26 | >0.9999 | 0.703 | 0.11 | 0.3562 | 2.101 | 0.43 | >0.9999 | 0.353 | 0.13 |

| Lipids | BT *vs.* BL | | | BT *vs.* LL | | | BB *vs.* BL | | | BB *vs.* LL | | | BL *vs.* LL | | |
|---|---|---|---|---|---|---|---|---|---|---|---|---|---|---|---|
| | P-value | Z score | Effect size (r) | P-value | Z score | Effect size (r) | P-value | Z score | Effect size (r) | P-value | Z score | Effect size (r) | P-value | Z score | Effect size (r) |
| **IgA** | | | | | | | | | | | | | | | |
| CL | 0.7028 | 1.810 | 0.35 | 0.2349 | 2.265 | 0.60 | >0.9999 | 1.503 | 0.29 | 0.4350 | 2.019 | 0.52 | >0.9999 | 0.994 | 0.17 |
| PE | >0.9999 | 1.633 | 0.32 | 0.9453 | 1.672 | 0.44 | >0.9999 | 0.864 | 0.16 | >0.9999 | 0.950 | 0.24 | >0.9999 | 0.236 | 0.04 |
| PI | 0.2623 | 2.223 | 0.43 | 0.1009 | 2.573 | 0.68 | 0.5994 | 1.881 | 0.36 | 0.2270 | 2.279 | 0.58 | >0.9999 | 0.849 | 0.14 |
| PTC | 0.4312 | 2.023 | 0.29 | 0.2559 | 2.232 | 0.59 | >0.9999 | 0.827 | 0.15 | >0.9999 | 1.132 | 0.29 | >0.9999 | 0.579 | 0.09 |
| SL | 0.3649 | 2.091 | 0.41 | 0.0949 | 2.594 | 0.69 | 0.5466 | 1.922 | 0.37 | 0.1323 | 2.478 | 0.64 | >0.9999 | 1.107 | 0.19 |
| **IgM** | | | | | | | | | | | | | | | |
| CL | >0.9999 | 0.850 | 0.16 | >0.9999 | 1.413 | 0.37 | >0.9999 | 0.255 | 0.04 | >0.9999 | 0.920 | 0.23 | >0.9999 | 1.087 | 0.18 |
| PE | >0.9999 | 1.500 | 0.29 | 0.4386 | 2.015 | 0.53 | 0.7361 | 1.789 | 0.34 | 0.1973 | 2.331 | 0.60 | >0.9999 | 1.070 | 0.18 |
| PI | >0.9999 | 0.095 | 0.01 | >0.9999 | 0.503 | 0.13 | >0.9999 | 1.137 | 0.21 | 0.8607 | 1.716 | 0.44 | >0.9999 | 1.054 | 0.18 |
| PTC | >0.9999 | 0.677 | 0.13 | >0.9999 | 1.497 | 0.40 | >0.9999 | 0.378 | 0.07 | >0.9999 | 0.607 | 0.15 | >0.9999 | 1.526 | 0.26 |
| SL | >0.9999 | 1.256 | 0.24 | 0.3905 | 2.064 | 0.55 | >0.9999 | 1.119 | 0.21 | 0.4336 | 2.020 | 0.52 | >0.9999 | 1.564 | 0.26 |
| **IgG** | | | | | | | | | | | | | | | |
| CL | >0.9999 | 0.250 | 0.04 | >0.9999 | 0.952 | 0.25 | >0.9999 | 0.293 | 0.05 | >0.9999 | 1.598 | 0.41 | 0.3475 | 2.111 | 0.36 |
| PE | 0.2717 | 2.209 | 0.43 | 0.0221 | 3.061 | 0.81 | >0.9999 | 1.447 | 0.27 | 0.1490 | 2.435 | 0.63 | 0.8201 | 1.739 | 0.29 |
| PI | 0.2491 | 2.243 | 0.44 | 0.0405 | 2.874 | 0.76 | 0.4203 | 2.033 | 0.39 | 0.0624 | 2.735 | 0.70 | >0.9999 | 1.351 | 0.23 |
| PTC | >0.9999 | 1.318 | 0.25 | >0.9999 | 0.662 | 0.17 | >0.9999 | 0.797 | 0.15 | >0.9999 | 1.386 | 0.35 | >0.9999 | 1.029 | 0.17 |
| SL | 0.6434 | 1.850 | 0.36 | 0.0682 | 2.705 | 0.72 | >0.9999 | 1.598 | 0.30 | 0.1060 | 2.556 | 0.66 | 0.8743 | 1.709 | 0.29 |

IgA = immunoglobulin A; IgM = immunoglobulin M; IgG = immunoglobulin G; CL = cardiolipin; PE = phosphatidylethanolamine; PI = phosphatidylinositol; PTC = phosphatidylcholine; SL = sulfatide; TT = tuberculoid leprosy; BT = borderline tuberculoid; BB = borderline borderline; BL = borderline lepromatous; LL = lepromatous leprosy; IQR = interquartile range; *Kruskal-Wallis test with Dunn's multiple comparison; **Effect size: small if r=0.10, medium if r=0.30, and large if r=0.50.

**Supplementary Table S5A** - Values of lipid antibodies in patients affected by leprosy stratified by Madrid classification.

| Lipids | IND | TT | BB | LL | P-value* |
|---|---|---|---|---|---|
| | median (IQR 25–75%). pg/mL | | | | |
| **IgA** | | | | | |
| CL | 0.9940 (0.7585–1.0920) | 0.8795 (0.7119–1.2740) | 1.0390 (0.7027–1.2870) | 1.5170 (0.8826–1.8310) | 0.2923 |
| PE | 1.1410 (0.8997–1.2920) | 1.0310 (0.9821–1.3360) | 1.1220 (0.9381–1.4000) | 1.4220 (0.8210–1.9850) | 0.7025 |
| PI | 0.6886 (0.4312–0.9417) | 0.8121 (0.3560–1.1140) | 0.7490 (0.4769–1.0540) | 1.0780 (0.6790–1.4330) | 0.3117 |
| PTC | 0.8008 (0.6632–0.9328) | 0.7151 (0.6532–0.8452) | 0.9078 (0.5753–1.1680) | 1.1830 (0.6570–1.7110) | 0.1907 |
| SL | 0.3940 (0.3226–0.5769) | 0.4461 (0.3233–0.7101) | 0.5643 (0.3110–0.7612) | 0.7792 (0.4888–1.0030) | 0.0842 |
| **IgM** | | | | | |
| CL | 0.1670 (0.07923–0.2165) | 0.1665 (0.0974–0.2758) | 0.1910 (0.1250–0.2900) | 0.2880 (0.1090–0.6190) | 0.2089 |
| PE | 0.3491 (0.2275–0.5172) | 0.4218 (0.2800–0.5240) | 0.3540 (0.2716–0.5540) | 0.5125 (0.2960–0.7590) | 0.2761 |
| PI | 0.4261 (0.1753–0.5573) | 0.3032 (0.2379–0.4297) | 0.3077 (0.2701–0.3912) | 0.3678 (0.2611–0.5858) | 0.5435 |
| PTC | 0.3408 (0.1726–0.4025) | 0.2990 (0.2131–0.4643) | 0.2906 (0.2159–0.3910) | 0.3463 (0.2544–0.6178) | 0.4452 |
| SL | 0.2982 (0.2220–0.4239) | 0.3136 (0.2447–0.4086) | 0.3171 (0.2588–0.3771) | 0.4987 (0.2592–0.6077) | 0.1790 |
| **IgG** | | | | | |
| CL | 0.5092 (0.4078–1.0970) | 0.5445 (0.3502–0.7141) | 0.4927 (0.3530–0.6069) | 0.7033 (0.4670–0.9120) | 0.1901 |
| PE | 0.2388 (0.1967–0.2779) | 0.2706 (0.2223–0.3394) | 0.2935 (0.2329–0.3730) | 0.3938 (0.3526–0.5790) | 0.0075 |
| PI | 0.2619 (0.1996–1.4550) | 0.8023 (0.2437–1.7380) | 0.3672 (0.2540–1.6540) | 1.6050 (1.282–2.1270) | 0.0493 |
| PTC | 0.5548 (0.4789–0.7466) | 0.8061 (0.5980–1.7350) | 0.7516 (0.5943–1.1140) | 0.9557 (0.6934–1.3290) | 0.1228 |
| SL | 0.6372 (0.5602–0.7971) | 0.9270 (0.6937–1.0610) | 0.8462 (0.7124–1.0510) | 1.1170 (0.9042–1.6250) | 0.0050 |

IgA = immunoglobulin A; IgM = immunoglobulin M; IgG = immunoglobulin G; CL = cardiolipin; PE = phosphatidylethanolamine; PI = phosphatidylinositol; PTC = phosphatidylcholine; SL = sulfatide; IND = indeterminate leprosy; TT = tuberculoid leprosy; BB = borderline borderline; LL = lepromatous leprosy; IQR = interquartile range; *Kruskal-Wallis test.

**Supplementary Table S5B** - Statistical metrics for lipid antibodies in patients affected by leprosy stratified by Madrid classification.

| Lipids | IND *vs.* TT | | | IND *vs.* BB | | | IND *vs.* LL | | |
|---|---|---|---|---|---|---|---|---|---|
| | P-value* | Z score | Effect size (r)** | P-value* | Z score | Effect size (r)** | P-value* | Z score | Effect size (r)** |
| **IgA** | | | | | | | | | |
| CL | >0.9999 | 0.184 | 0.04 | >0.9999 | 0.438 | 0.07 | 0.7750 | 1.517 | 0.36 |
| PE | >0.9999 | 0.077 | 0.01 | >0.9999 | 0.412 | 0.06 | >0.9999 | 0.836 | 0.20 |
| PI | >0.9999 | 0.038 | 0.009 | >0.9999 | 0.230 | 0.03 | >0.9999 | 1.335 | 0.32 |
| PTC | >0.9999 | 0.417 | 0.09 | >0.9999 | 0.624 | 0.10 | >0.9999 | 1.305 | 0.31 |
| SL | >0.9999 | 0.310 | 0.07 | >0.9999 | 0.898 | 0.14 | 0.2029 | 2.122 | 0.51 |
| **IgM** | | | | | | | | | |
| CL | >0.9999 | 0.533 | 0.12 | >0.9999 | 1.102 | 0.18 | 0.3407 | 1.905 | 0.46 |
| PE | >0.9999 | 0.601 | 0.14 | >0.9999 | 0.529 | 0.08 | 0.5691 | 1.670 | 0.40 |
| PI | >0.9999 | 0.441 | 0.10 | >0.9999 | 0.457 | 0.07 | >0.9999 | 0.549 | 0.13 |
| PTC | >0.9999 | 0.174 | 0.04 | >0.9999 | 0.013 | 0.002 | >0.9999 | 1.114 | 0.27 |
| SL | >0.9999 | 0.135 | 0.03 | >0.9999 | 0.247 | 0.04 | 0.6336 | 1.618 | 0.39 |
| **IgG** | | | | | | | | | |
| CL | >0.9999 | 0.339 | 0.07 | >0.9999 | 0.781 | 0.13 | >0.9999 | 0.806 | 0.19 |
| PE | >0.9999 | 1.058 | 0.24 | 0.5738 | 1.666 | 0.27 | 0.0093 | 3.165 | 0.76 |
| PI | >0.9999 | 0.805 | 0.18 | >0.9999 | 1.115 | 0.18 | 0.0735 | 2.505 | 0.60 |
| PTC | 0.3914 | 1.844 | 0.43 | 0.3796 | 1.857 | 0.30 | 0.1055 | 2.374 | 0.57 |
| SL | 0.3829 | 1.853 | 0.43 | 0.1902 | 2.148 | 0.35 | 0.0027 | 3.511 | 0.85 |

| Lipids | TT *vs.* BB | | | TT *vs.* LL | | | BB *vs.* LL | | |
|---|---|---|---|---|---|---|---|---|---|
| | P-value* | Z score | Effect size (r)** | P-value* | Z score | Effect size (r)** | P-value* | Z score | Effect size (r)** |
| **IgA** | | | | | | | | | |
| CL | >0.9999 | 0.304 | 0.04 | 0.6261 | 1.624 | 0.33 | 0.6202 | 1.629 | 0.25 |
| PE | >0.9999 | 0.653 | 0.10 | >0.9999 | 1.110 | 0.23 | >0.9999 | 0.680 | 0.10 |
| PI | >0.9999 | 0.358 | 0.05 | 0.5701 | 1.670 | 0.34 | 0.6183 | 1.630 | 0.25 |
| PTC | 0.9180 | 1.429 | 0.22 | 0.2214 | 2.087 | 0.43 | >0.9999 | 1.087 | 0.17 |
| SL | >0.9999 | 0.721 | 0.11 | 0.1633 | 2.208 | 0.46 | 0.3321 | 1.916 | 0.29 |
| **IgM** | | | | | | | | | |
| CL | >0.9999 | 0.661 | 0.10 | 0.5613 | 1.677 | 0.35 | >0.9999 | 1.344 | 0.20 |
| PE | >0.9999 | 0.187 | 0.02 | >0.9999 | 1.310 | 0.27 | 0.4982 | 1.733 | 0.27 |
| PI | >0.9999 | 0.046 | 0.007 | >0.9999 | 1.197 | 0.24 | >0.9999 | 1.372 | 0.21 |
| PTC | >0.9999 | 0.238 | 0.03 | >0.9999 | 1.145 | 0.23 | 0.6741 | 1.588 | 0.24 |
| SL | >0.9999 | 0.125 | 0.01 | 0.4266 | 1.805 | 0.37 | 0.2627 | 2.016 | 0.31 |
| **IgG** | | | | | | | | | |
| CL | >0.9999 | 0.525 | 0.08 | 0.9919 | 1.387 | 0.28 | 0.1882 | 2.152 | 0.33 |
| PE | >0.9999 | 0.633 | 0.09 | 0.0590 | 2.581 | 0.53 | 0.0874 | 2.443 | 0.38 |
| PI | >0.9999 | 0.281 | 0.04 | 0.2248 | 2.081 | 0.43 | 0.1705 | 2.191 | 0.34 |
| PTC | >0.9999 | 0.267 | 0.04 | >0.9999 | 0.678 | 0.14 | >0.9999 | 1.062 | 0.16 |
| SL | >0.9999 | 0.099 | 0.01 | 0.2428 | 2.049 | 0.42 | 0.1188 | 2.330 | 0.36 |

IgA = immunoglobulin A; IgM = immunoglobulin M; IgG = immunoglobulin G; CL = cardiolipin; PE = phosphatidylethanolamine; PI = phosphatidylinositol; PTC = phosphatidylcholine; SL = sulfatide; IND = indeterminate leprosy; TT = tuberculoid leprosy; BB = borderline borderline; LL = lepromatous leprosy; IQR = interquartile range. *Kruskal-Wallis test with Dunn's multiple comparison; **Effect size: small if r=0.10, medium if r=0.30, and large if r=0.50.
